# Use of Temporary Implantable Biomaterials to Reduce Leg Pain and Back Pain in Patients with Sciatica and Lumbar Disc Herniation

**DOI:** 10.3390/ma3053331

**Published:** 2010-05-19

**Authors:** Gere S. diZerega, Melissa M. Traylor, Lisa S. Alphonso, Samuel J. Falcone

**Affiliations:** 1 Keck School of Medicine at University of Southern California / 1321 N. Mission Road, Los Angeles, CA 90033, USA; 2 FzioMed, Inc. / 231 Bonetti Drive, San Luis Obispo, CA 90033, USA; E-Mails: mtraylor@fziomed.com (M.M.T.); lalphonso@fziomed.com (L.S.A.); sfalcone@fziomed.com (S.J.F.)

**Keywords:** biomaterial, viscoelastic gel, back pain, sciatica, lumbar surgery, fibrosis, cytokines, disc herniation, oxiplex, healon

## Abstract

The principle etiology of leg pain (sciatica) from lumbar disc herniation is mechanical compression of the nerve root. Sciatica is reduced by decompression of the herniated disc, *i.e.*, removing mechanical compression of the nerve root. Decompression surgery typically reduces sciatica more than lumbar back pain (LBP). Decompression surgery reduces mechanical compression of the nerve root. However, decompression surgery does not directly reduce sensitization of the sensory nerves in the epidural space and disc. In addition, sensory nerves in the annulus fibrosus and epidural space are not protected from topical interaction with pain mediators induced by decompression surgery. The secondary etiology of sciatica from lumbar disc herniation is sensitization of the nerve root. Sensitization of the nerve root results from a) mechanical compression, b) exposure to cellular pain mediators, and/or c) exposure to biochemical pain mediators. Although decompression surgery reduces nerve root compression, sensory nerve sensitization often persists. These observations are consistent with continued exposure of tissue in the epidural space, including the nerve root, to increased cellular and biochemical pain mediators following surgery. A potential contributor to lumbar back pain (LBP) is stimulation of sensory nerves in the annulus fibrosus by a) cellular pain mediators and/or b) biochemical pain mediators that accompany annular tears or disruption. Sensory fibers located in the outer one-third of the annulus fibrosus increase in number and depth as a result of disc herniation. The nucleus pulposus is comprised of material that can produce an autoimmune stimulation of the sensory nerves located in the annulus and epidural space leading to LBP. The sensory nerves of the annulus fibrosus and epidural space may be sensitized by topical exposure to cellular and biochemical pain mediators induced by lumbar surgery. Annulotomy or annular rupture allows the nucleus pulposus topical access to sensory nerve fibers, thereby leading to LBP. Coverage of the annulus and adjacent structures in the epidural space by absorbable viscoelastic gels appears to reduce LBP following surgery by protecting sensory fibers from cellular and biochemical pain mediators.

## 1. Introduction

Patients with sciatica and severe lumbar back pain (LBP) resulting from lumbar disc herniation comprise a clinically challenging subgroup of patients [1,2,3]. Appendix table 1 summarizes many of the clinical reports that have appeared throughout the medical literature over many years. Most recently, the large, multicenter, NIH-funded SPORT study confirmed that most patients with sciatica from a herniated lumbar disc also have lumbar back pain [4,5]. This review summarizes preclinical and clinical data that provide information regarding the source of LBP in these patients. A hypothesis is developed which may provide direction for the development of surgical procedures and locally applied devices to reduce the post operative LBP which often accompanies successful reduction of sciatic pain following removal of the herniated disc.

Many types of biomaterials have been implanted in the epidural space in an effort to reduce postoperative pain caused by scar formation. None of these have been integrated into lumbar spine surgery as standard practice due to the challenges presented by the biomaterials as well as the unique anatomical space. Massie *et al.* concluded that one potential mode of action for the reduction of pain following surgery with the use of viscoelastic gels is the decreased migration of inflammatory cells into the epidural space by the viscous environment of the gel [6]. Viscoelastic gels would provide a protective tissue coating that decreases fibrosis and shields the nociceptors present on the exposed sensory nerves from pain mediators. The utilization of a mechanical barrier that coats and separates tissues in the lumbar spine provides some measure of surface protection of the sensory nerve against inflammatory mediators that occur as a result of surgery as well as outpouring from the annulotomy site itself.

diZerega *et al.* reported on a modern biomaterial for adhesion prevention which can be formulated into a flowable, biologically inert, viscoelastic gel with tissue adherence appropriate for use in minimally invasive surgery [7]. The device coats surgically traumatized tissues and remains at the site of placement even in gravitationally dependent areas. The data demonstrate that polysaccharide gels that coat healing tissues protect the tissues from cellular and biochemical pain mediators and fibrotic bridges that lead to adhesions during the healing process. The results of these studies demonstrated that the gels separated tissues during healing, thereby reducing their interaction by the interposition of a barrier.

## 2. Discussion

### 2.1. Sciatica

Decompression surgery for disc herniation typically improves sciatica more than lumbar back pain [4,8]. Sciatica is reduced by removing compression on the exiting root of the sciatic nerve. Decompression further reduces the sensitization of the nerve root to pain mediators by reducing inflammation caused by mechanical pain stimulation [9].

There are several sources of pain generation in disc injury involving an intervertebral disc that is degenerative, bulging, or protruding [10,11,12]. Disc herniation provides direct pressure by disc tissue on the nerve root. Mechanical compression of a nerve alone is not necessarily painful, however, if that nerve is inflamed (irritated, tender, swollen), it can produce severe pain with a small amount of mechanical compression. Nerve root compression is an important factor in generating inflammation and resultant sciatica [13,14,15]. When both nerve compression and inflammation around the nerve root are present, there is more nerve injury and pain perception than after either event alone [16,17].

Spinal nerve root compression does not cause sciatica in all circumstances because more than 50% of “normal,” asymptomatic people who have disc prolapses compressing the nerve roots have no pain [18]. In symptomatic individuals, the nerves are sensitized to compression, probably by biochemical pain mediators [19]. The inflammatory response that occurs as a result of nerve root compression also affects the sensory components of the lower back including the sensory nerves of the adjacent soft tissue. The inflammatory process is believed to sensitize the nerve root to all incoming stimuli. In such a state, even minor mechanical stimulation of the nerve root can evoke severe back pain. These pain mediators interact topically with nociceptors. Limiting the direct interaction of pain mediators with nociceptors was shown to reduce pain in preclinical models [6,20]. 

The mechanical compression of the nerve root may also lead to a series of intraneural tissue reactions, including edema, demyelination, and fibrosis that sensitize the surface of the nerve to pain mediators [9,13,21,22]; or tether the nerve root to adjacent tissues [23,24]. Mechanical compression increases microvascular permeability of the endoneural capillaries resulting in inflammation within the nerve root. Sensitizing the nerve root by topical exposure to pain mediators contributes to the pathogenesis of sciatica [13].

### 2.2. Lumbar Back Pain

The intervertebral disc is the main source of lumbar back pain (LBP). Intraoperative findings under local anesthesia showed that LBP was reproduced by stimulation of the outer annulus or the posterior longitudinal ligament (locations of sensory neurons). In contrast, sciatica was induced by mechanical stimulation of nerve roots [25,26,27,28,29,30].

The reduction in LBP that follows decompression surgery results from reduced production of pain mediators (biochemical as well as cellular) in the epidural space (disc, adjacent soft tissues, nerve root), which reduces stimulation of nociceptors in the sensory nerve fibers of the annulus and adjacent soft tissues. Decompression surgery also reduces the sensitization of these sensory nerve fibers to stimulation by pain mediators [31]. 

Microtrauma damages the annulus fibrosus, allowing blood vessels and nerves a deeper penetration into the annulus fibrosus [21]. Increased vascular and neural in-growth are seen in discs associated with LBP. Malinsky demonstrated a variety of free nerve endings and some button-like terminals exist in the outer few layers of the lumbar annulus and noted partially and fully encapsulated mechanoreceptors confined to the annular surface [32,33]. These free nerve endings contribute to pain transmission from the disc producing LBP [32]. Furthermore, the concentration of nerves and blood vessels in the annulus increases with age [34].

Free nerve endings are present in the annulus fibrosus and epidural space (ligaments, nerve roots and muscles). In patients with sciatica caused by disc herniation, reports of LBP preceding sciatic pain are common. Patients with severe LBP associated with disc herniation and sciatica have greater density of sensory nerves in the annulus fibrosus and epidural space than patients with less severe LBP [13,16,21,35,36,37,38,39], which results in sensitization of more sensory nerves and additional LBP following decompression surgery. It has been suggested that this pain may be caused by topical stimulation of nerve endings in the annulus fibrosus as a result of an annular tear and/or herniation and later by inflammation associated with extrusion of the nucleus pulposus. Chemical sensitization of sensory nerve fibers of the disc is induced by inflammation caused initially by disc herniation and later by the trauma of surgical decompression. Removing the herniating portion of the disc and/or the residual nucleus pulposus reduces inflammation in the epidural space [10,12,40,41,42]. Trauma to an intervertebral disc may damage disc components, resulting in the production of irritants (biochemical mediators), which may drain either into the spinal canal, irritating nerves, or into the vertebral body, setting up an autoimmune reaction resulting in LBP [10,12,21,43,44]. 

Biochemical mediators of pain are present in disc herniation tissue [10]. Axonal injuries and inflammatory stimulation of nociceptors alter nerve root excitability and thereby play an important role in LBP [45,46]. Local production of chemokines within the epidural space also contributes to LBP [47].

A number of experimental studies demonstrated the negative affects of disc tissue, and in particular the nucleus pulposus, on nerve roots [48,49,50,51,52,53,54,55]. When the substance of the nucleus pulposus comes into contact with sensory nerves of the epidural tissues including the outer annulus it:

1) induces degeneration of nerve fibers;
2) increases discharge of nerve fibers;
3) attracts inflammatory cells (cellular mediators of pain) and;
4) induces increased intraneural capillary permeability.

### 2.3. Lumbar Disc: Anatomy

**Figure 1:**
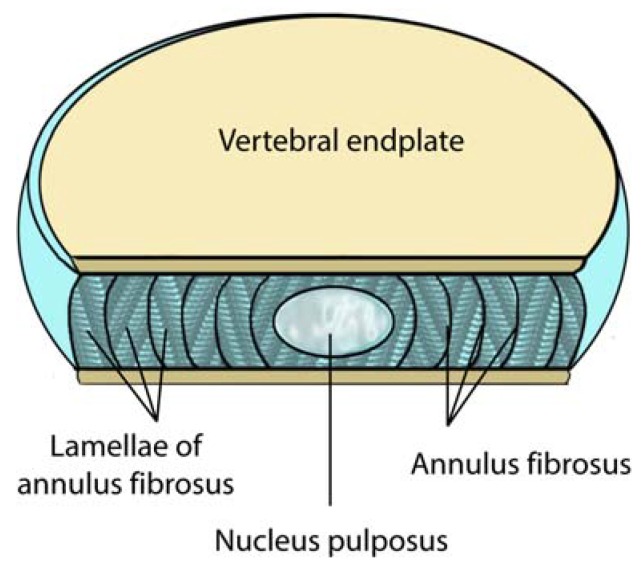
A sectional view of a normal lumbar disc. Note the locations of the nucleus pulposus, the vertebral end plate and the annulus fibrosus. The intervertebral lumbar disc is typically 4cm wide and 7-10 mm thick (adapted from Raj *et al.* [34]). The interior of the disc including the nucleus pulposus is avascular.

In order to understand how pain is generated in the disc and epidural space, a brief review of the disc, epidural space and associated neuroanatomy is useful. The disc is composed of a central nucleus pulposus surrounded peripherally by the annulus fibrosus (Figure 1). In normal young adults, the nucleus is a semi-fluid mass of mucoid material (glycosaminoglycans, proteoglycans, and collagen). The nucleus is composed of approximately 70-90% water in a young healthy disc, but this percentage generally decreases with age [9]. The annulus fibrosus consists of 10-20 concentric collagen fiber layers that surround the nucleus. The layers are arranged in alternating orientation of parallel fibers.

The vertebral endplate is a thin layer of cartilage located between the vertebral body and the intervertebral disc. Although normally composed of both hyaline and fibrocartilage in youth, older end plates are virtually entirely fibrocartilage exposing the sensory fibers that course through these areas to topical stimulation. The intervertebral disc is the largest avascular structure in the body. As a result, exposure of epidural tissues to the nucleus pulposus produces an autoimmune response increasing the concentration of cellular and biochemical pain mediators.

The central component to any injury involving the lumbosacral discs is the natural aging process and/or trauma [56]. Disc aging typically involves circumferential tears or fissures in the outer annulus. These changes are thought to result from repetitive microtrauma. Brown *et al.* and Ohtori *et al.* reported that, in patients with lumbar back pain (LBP), there were increases in the density of sensory nerve fibers in the endplates and defects in the endplate cartilage, strongly suggesting that the endplates and vertebral bodies were sources of pain [57,58].

**Figure 2:**
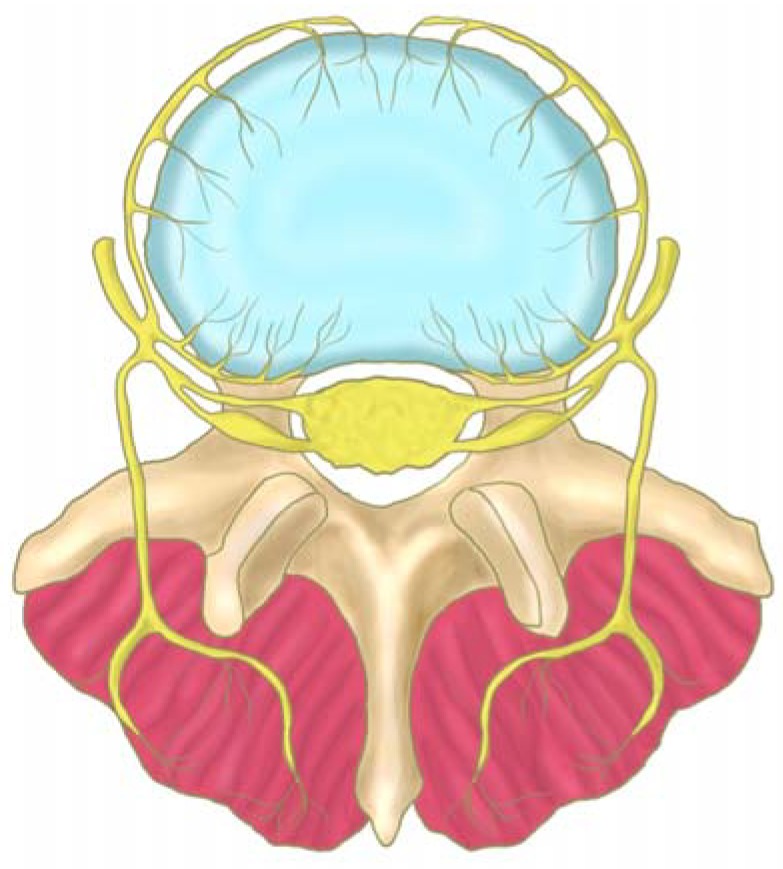
The course of the sinu-vertebral nerve (yellow), which innervates the postero-lateral region of the disc (blue). The nerve exits from the nerve root and enters the vertebral foramen, where it divides into a major ascending and a lesser descending branch (adapted from Raj *et al.* [34]).

Sensory nerves in the disc often accompany blood vessels present in the longitudinal ligaments adjacent to the disc, but they can also occur independently, arising as branches of the sinuvertebral nerve or derived from the ventral rami or gray rami communicans [34] (Figure 2). The ventral rami and gray rami communicans form a ventral plexus that supplies the anterior and lateral aspects of the annulus and the anterior longitudinal ligament. The posterior annulus fibrosus and the posterior longitudinal ligament are innervated by the sinuvertebral nerve (contributes branches to the dorsal plexus), which consists of sensory fibers. Each sinuvertebral nerve supplies the disc at its level of entry into the vertebral canal [59,60]. Most of the nerve fibers are sensory in origin and are involved in nociception [33,39,60,61,62].

**Figure 3:**
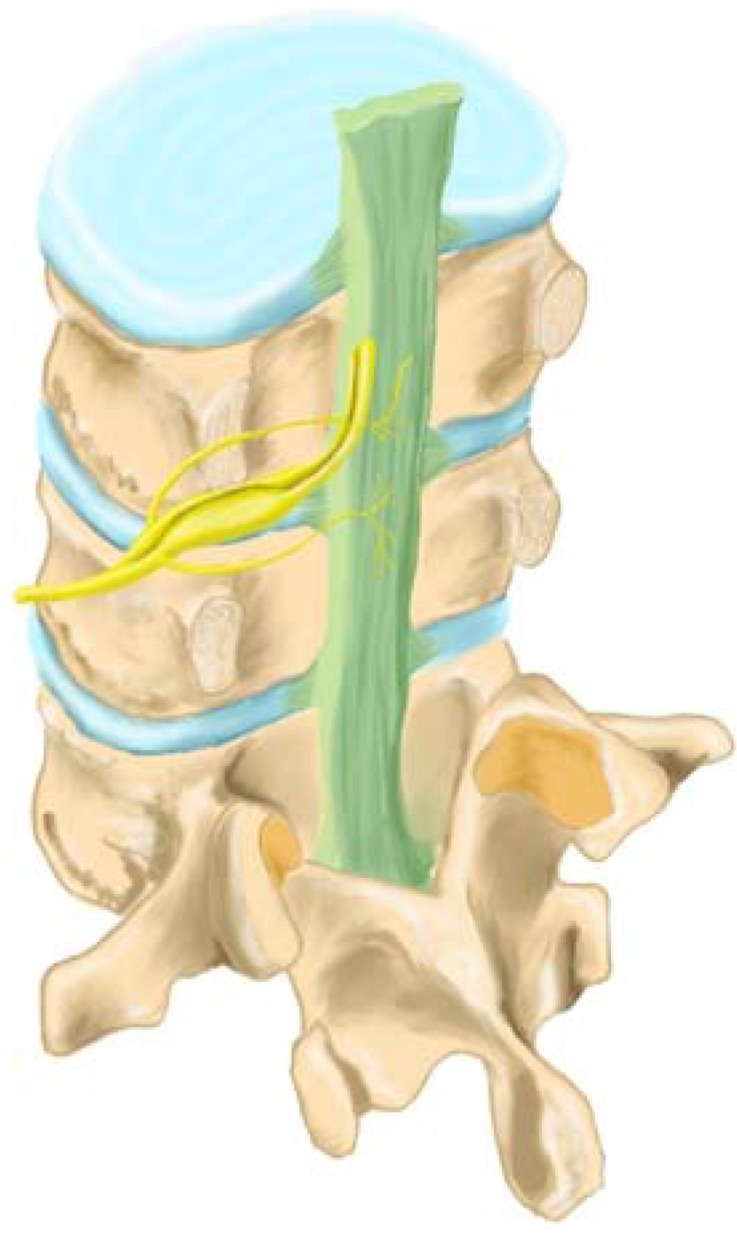
Sensory innervation of the posterior longitudinal ligament (green) and the disc annulus (blue) occurs by the ascending branch of the sinuvertebral nerve (yellow) (adapted from Raj *et al.* [34]).

The anterior longitudinal ligament also receives sensory innervation from branches that originate in the nerve root. The posterior longitudinal ligament (PLL) is richly innervated by nociceptive fibers from the ascending branch of the sinuvertebral nerve (Figure 3). These nerves also provide sensory innervation of the adjacent outer layers of the annulus fibrosus [34]. Herniation and rupture of the disc/longitudinal ligament typically leads to exposure of the epidural space to pain mediators.

### 2.4. Sensory Innervation of the Disc

In 1949, Herlihy reported that direct innervation of the intervertebral disc could lead to intrinsic pain stimulation at the disc [59,63]. Subsequently, multiple studies conclusively demonstrated that the intervertebral disc is innervated [59,64]. In a normal disc, the outer one third of the annulus is innervated; the inner two thirds of the annulus and the nucleus pulposus receive no innervation [59]. A high proportion of nociceptive nerve fibers arising from the annulus of the lumbar discs pass through the sympathetic trunks as sympathetic sensory afferents. These pain receptors are sensitized by changes in external pressure (mechanoreceptors) or inflammatory irritation [22,65,66,67]. 

The lumbar intervertebral disc is innervated by the sinuvertebral nerves (Figure 3) consisting of spinal sensory fibers and postganglionic sympathetic fibers [25,58,68,69,70]. Sensory neurons involved in pain perception (nociceptive) relating to inflammatory pain as occurs with disc herniation or following decompression surgery are typically small, peptide-containing neurons [58,70,71]. As a result, peripheral nerve injuries can lead to pain sensations that are expressed within minutes, days, weeks or months following the actual traumatic event (for example disc herniation or surgical anulotomy) [72,73,74]. Many painful lumbar neuropathies involve subtotal nerve damage including decompression surgery [75]. Patients with painful neuropathies suffer from both spontaneous pain (allodynia) and from a variety of different types of abnormal evoked-pain sensations (hyperalgesia). Application of biochemical pain mediators to the surface of the nerve root produce hyperalgesia [75,76]. 

**Figure 4:**
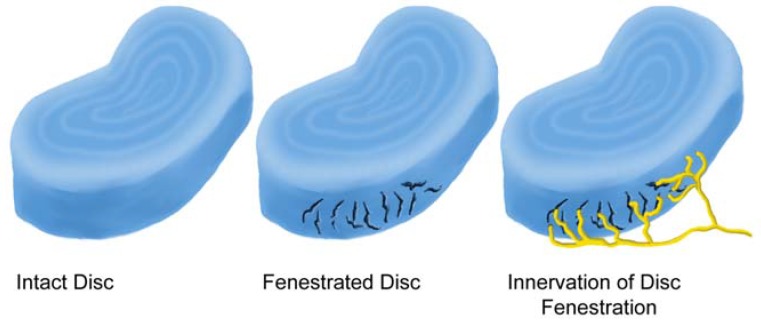
Fenestrations caused by trauma and/or aging are often the site of nerve ingrowth.

**Figure 5:**
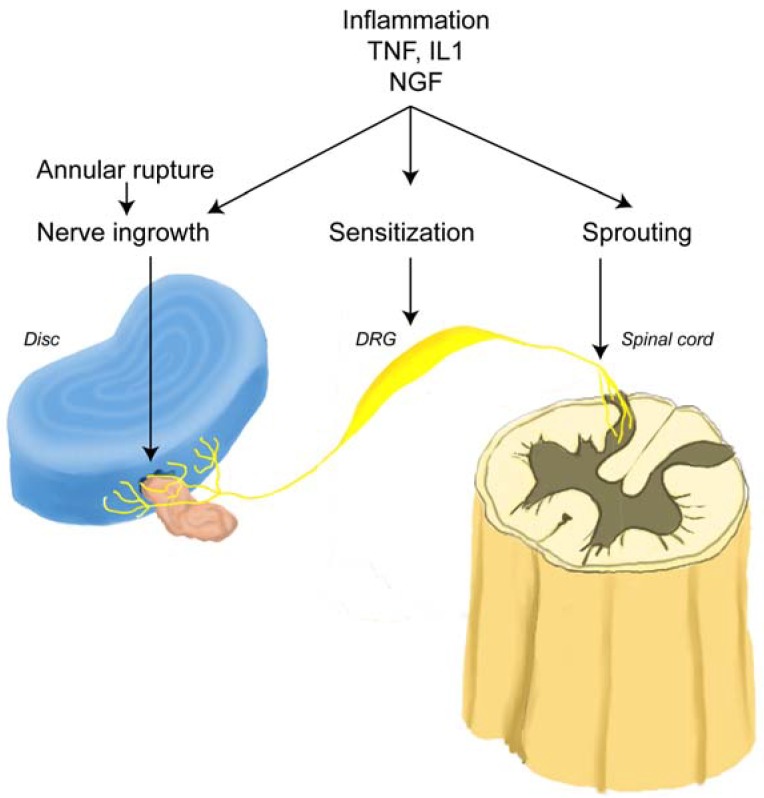
Schematic representation of a mechanism for LBP. An annular rupture leads to extrusion of the nucleus pulposus (pink) outside annulus fibrosus (blue), which induces nerve injury and nerve in-growth into the disc (yellow). Under inflammatory conditions which may occur following surgery, nerve growth factor (NGF) is induced in the disc and acts on the nerve root (DRG) through the peripheral terminals in the disc. Disc-innervating neurons have a high sensitivity to NGF. NGF may promote nerve in-growth into the disc, sensitize DRG neurons, and cause neuronal sprouting into the dorsal horn (adapted from Takahashi *et al.* [38]).

The innervation of the disc is concentrated in the outermost part of the annulus fibrosus and endplate [39,68,77,78,79]. Palmgren demonstrated sensory nerve terminals in herniated lumbar disc tissue, in the periphery of the annulus fibrosus, and along deeper annular tears [78]. Ashton *et al.* and Aoki *et al.* identified nerve structures in lumbar discs from asymptomatic patients extending 3 mm into the annulus fibrosus [35,71]. In contrast, disc material obtained from patients with LBP showed deeper in-growth of blood vessels and nerves. Freemont examined the innervation of the inner disc using 46 biopsy samples (30 from levels with pain and 16 from levels with no pain) [21]. Innervation of the inner disc was observed more frequently in painful discs than in asymptomatic discs. They further demonstrated the presence of nerve fibers in painful discs demonstrating that nociceptive nerve fibers were growing into the painful disc. Nerve in-growth into the inner disc follows the development of fenestrations resulting from trauma and/or aging (Figure 4). These sensory afferents, which can transmit pain from the disc itself, contribute to LBP with herniation (Figure 5).

The pain receptors of the intervertebral discs are nociceptors, which are activated under inflammatory conditions when their surface comes into contact with biochemical and cellular mediators of pain, which lead to pain perception. Inflammatory changes may cause the silent nociceptors to become responsive to mechanical stimuli, and this nociceptive information is perceived as LBP [27,80]. Takebayashi found that the lumbar intervertebral discs were not responsive to mechanical stimulation under normal conditions, but once inflamed by the topical application of biochemical pain mediators, mechanically insensitive afferents responded to mechanical stimulation [81].

Freemont *et al.* demonstrated nerves in the annulus with the morphology of nociceptors [21]. Inflammatory cells, mostly macrophages, were found in disc herniation tissue, indicating a topical inflammatory reaction. In contrast, only a few macrophages were observed in normal disc tissue [82]. Histamine produced by macrophages, prostaglandin E_2_ [82,83,84] and bradykinin all act as chemical mediators of pain. These mediators also sensitize the peripheral nerve endings that evoke pain sensation [85]. The neural structures are susceptible to stimulation by pressure or chemical mediators produced as a result of herniation [79].

### 2.5. Sensory Innervation of the Epidural Space

The soft tissues (ligaments, muscles) and nerve root adjacent to the disc contribute to lumbar back pain (LBP) by stimulation of sensory nerves [65,86]. Rupture of the posterior longitudinal ligament and extrusion of nucleus pulposus into the epidural space (Figure 5) evokes an autoimmune reaction and infiltration of cellular pain mediators [41,87,88,90]. Chemical mediators throughout the epidural space (especially from the intervertebral disc itself) are a significant source of both LBP and sciatica as well as sensitization of sensory nerves [11,91,92,93]. Burke *et al.* found that patients with severe LBP generally had higher levels of inflammatory mediators than those patients with lower levels of disc pain [10]. 

### 2.6. Sensitization of Sensory Nerves

Mechanical stimuli which are normally innocuous to disc nociceptors can generate an amplified response which has been termed ‘peripheral sensitization’ [47]. This may explain why some herniating discs are painful and others are not [26]. Exposed nuclear material is known to irritate the spinal nerve root and the sinuvertebral nerve endings [26,55,94,95]. In herniated discs the inflammatory granulation tissue present in annular tears and containing sensory nerves [21,44,47,62] behaves in a similar way [10]. This peripheral sensitization has been confirmed clinically [96]. Increased numbers of mechanoreceptors [97] and sensory neurons are found in discs from patients with lumbar back pain (LBP) [57,98].

Exposure of epidural tissue to inflammatory cytokines from the disc nucleus results in both nerve sensitization [12] and nerve injury [42,84]. Franson showed that human PLA2 concentration in the intervertebral disc are 20-10,000 times higher than the PLA2 found in other human tissues [99]. A wide variety of pain mediators that come in contact with the nerve root during and after disc surgery can sensitize neural tissue to postoperative pain and neurological symptoms [10,11,12,19,100] Increase in sensory nerve excitability that can occur following decompression surgery often prolongs sensory nerve sensitization resulting in pain and hyperalgesia long after the surgical procedure [101]. 

The combination of mechanical compression (mass effect of herniated disc) and chemical irritation by cellular and biochemical mediators (inflammation around nerve root) induces more LBP than either factor alone [16]. As reviewed by Cohen *et al.*, the concept of chemical sensitization may explain the contrasting responses between LBP and asymptomatic discs. Tumor necrosis factor-α (TNF-α) is expressed in the nucleus pulposus and plays a role in generating sciatic pain in patients with disc herniation [102,103,104]. Interleukin-1β (IL-1 β), which is produced in tissues involved in disc herniation, has the capacity to produce hyperalgesia. Also, NGF, which is up regulated by such mediators, has a sensitizing effect on nerve fibers. The levels of inflammatory mediators are higher in painful discs than in asymptomatic discs, suggesting that their interaction with tissues in the epidural space would produce LBP [10,105].

### 2.7. Post Operative Pain

Surgical dissection and retraction cause nerve root trauma and cellular injury, expose neural tissues to blood and disc substance (nucleus pulposus), filling the epidural space with cellular and biochemical pain mediators. These lead to nerve root irritation, inflammation and fibrin production each of which can trigger additional inflammation, trauma, nerve root compression or tethering and sensory sensitization. Mechanical deformation of nerve tissue resulting from surgical dissection also contributes to prolonged postoperative lumbar back pain (LBP). Laminectomies may also induce an increase in the density of nociceptive neurons in the lumbar disc ascribable to axonal sprouting of fine sensory nerve fibers. Neuronal outgrowth of nociceptive afferents is associated with LBP after lumbar surgery [106]. The leaking "chemical soup" within the nucleus pulposus contains pain mediators that stimulate sensory fibers following annulotomy. Neuropeptides released from peripheral endings of nociceptive afferents are also inflammatory mediators and pain generators [107]. 

### 2.8. Pain Mediators during Herniation and Following Decompression Surgery

A large number of cells (principally inflammatory) and biochemicals are present in the disc and epidural space during herniation that are not present in normal disc tissue. They have been shown to interact with nociception and sensory nerve transmission and are collectively referred to here as “pain mediators”. The concentration of these pain mediators increase in these tissues following surgery. As a result, even though mechanical decompression is completed, pain often continues, especially lumbar back pain (LBP) (Appendix Table 2). 

### 2.9. Cellular Pain Mediators

#### 2.9.1. Neutrophils

Inflammatory cells at the surgical site increase in both number and activity. During and immediately following surgical injury, neutrophils enter the surgical field in large numbers. An increase in neutrophil number or concentration within the surgical site can contribute to pain. Neutrophils release a number of products that cause tissue destruction and continued inflammation (oxygen radicals, protease, *etc.*) that contribute to pain through topical interaction with sensory fibers and nociceptors [11,21,82,94,95,108]. 

#### 2.9.2. Macrophages

The second cell type to enter the epidural space after surgery is monocytes/macrophages. These cells secrete oxygen free-radicals, along with pro-inflammatory cytokines. Gronblad *et al.* reported high concentrations of macrophages and biochemical pain mediators in disc material obtained from patients with disc herniation [82]. Doita reported that mononuclear cells infiltrating along the margins of extruded discs expressed inflammatory mediators and appeared to induce neovascularization and persistence of inflammation [41,109]. Macrophages and mast cells are among the chief cellular mediators of inflammatory neuritis. Macrophages can produce a host of inflammatory molecules (e.g., interleukin 1 [110,111,112]) as well as tumor necrosis factor [110,111,113] and can also exert cytotoxic activity by direct physical contact or through the release of toxic by-products (e.g., nitric oxide [114] and proteases [115,116,117]. Macrophages also enhance vascular permeability, provide chemotactic signals and modulate inflammatory cell activities. Release of histamine induced by mast cell degranulation may play an important role in LBP [118]. Intraneural edema and the appearance of macrophages and other inflammatory cells occur at the site of compression leading to sensory nerve sensitization [13].

Haro *et al.* obtained similar data on the presence of macrophages in painful disc herniations. In addition, they were able to demonstrate statistically significant quantities of factor VII, monocyte chemotactic protein-1, and macrophage inflammatory protein-1 positive cells in symptomatic herniations [119]. Takahashi *et al.* demonstrated the presence of inflammatory cytokines in human tissue adjacent to nerve roots at the level of a symptomatic herniated disc removed at the time of surgery [120]. 

### 2.10. Biochemical Pain Mediators Identified in the Epidural Space in Patients with LBP

A large number of biochemical pain mediators have been identified in the disc and epidural space that contribute to LBP following decompression surgery (Appendix table 2).

Disc rupture/annulotomy exposes epidural tissues to the nucleus pulposus. The nucleus pulposus elicits an immune response by adjacent tissues in the epidural space [121]. Application of disc tissue to a nerve results in nerve fiber injury and pain [11,50,51,53,54,55]. TNF- α is released not only by the herniated disc, but is also released in cases of annular tear [44,122,123,124]. Takahashi reported that inflammatory cytokines were present in disc material removed from patients with herniated discs [120]. Herniated lumbar disc tissue from symptomatic patients contains elevated levels of TNF- α, nitric oxide (NO), prostaglandin E_2_ (PGE2), IL-1β, IL-6, IL-8, COX-2, and NOS [125,126] as compared with control disc tissue [41,127,128]. These biochemical mediators may be a direct stimulant of LBP as well as sensitize peripheral nociceptors [53,56].

**Figure 6:**
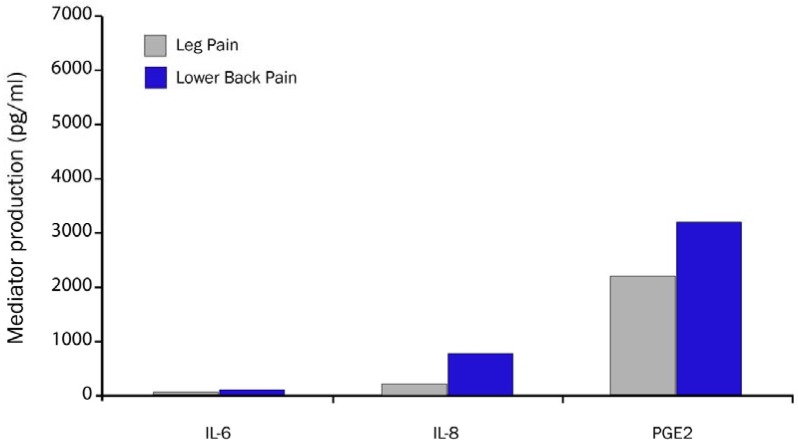
Biochemical mediator concentration is higher in disc from patients with LBP *vs.* patients with leg pain and LBP (adapted from Burke *et al.* [10]).

Burke *et al.* confirmed and extended these observations by quantitating proinflammatory biochemical pain mediators (IL-6, IL-8 and PGE2) in disc tissue obtained from patients undergoing lumbar surgery for sciatica and LBP (Figure 6) [10]. Those patients with LBP (n = 20) had significantly higher concentrations of biochemical pain mediators than patients with only sciatica (n = 63). Hyperalgesia is induced in an innervated nucleus pulposus by cellular and biochemical pain mediators. The exposure of the nucleus pulposus to the outer annulus fibrosus induces nerve injury. Hyperalgesic responses can be induced by contact of the nociceptors of sensory nerves with nucleus pulposus that signal LBP directly from the disc and epidural space [38]. 

### 2.11. Annular Leakage and Pain Sensitization

Disc herniation is typically preceded by one or more attacks of acute lumbar back pain (LBP) [43,129]. Moneta *et al.* demonstrated that peripheral annular tears in human are the principal source of LBP preceding herniation [130]. These findings were confirmed in clinical studies by Weinstein *et al.* and Hyodo *et al.*, and animal models by Murata *et al.* [5,129,131]. LBP may also occur when there is internal disruption of annular fibers but no tearing of the outer layers of the annulus. In this situation, there is local injury to the sensory fibers of the outer one-third of the annulus as well as leakage of the nucleus pulposus into the epidural space. Kayama *et al.* found that incision of the annulus fibrosus without disc herniation, but with a slow leakage of nucleus pulposus through the incision, induced significant pain and nerve damage in a dog model [37]. These preclinical studies supported their clinical experience that patients suffer from more severe pain when nucleus pulposus is communicating with the epidural space compared with those in whom the herniated nucleus pulposus is contained within the annulus fibrosus [44].

**Figure 7:**
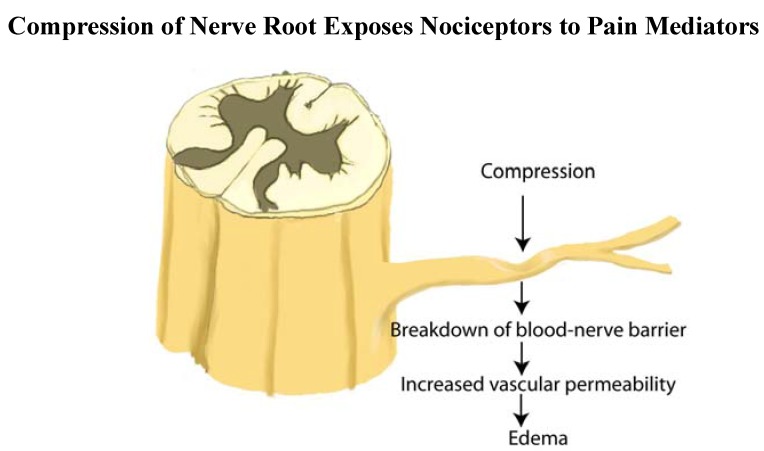
Nerve root compression produces increased vascular permeability at the site of compression, in the peripheral zone of a compressed anterior nerve root, and in the central zone of a compressed posterior nerve root. After nerve root compression, degeneration of the nerve is found in the area of increased vascular permeability. DR: dorsal root, DRG: dorsal root ganglion, VR: ventral root (adapted from Kobayashi *et al.* [13]).

Materials that leak from annular tears induce single level nerve root pain or multi-level nerve root pain through diffusion. Peng *et al.* found that the nerve injury caused by the combination of mechanical compression (mass effect of herniated nucleus pulposus) and chemical irritation (inflammation around the nerve root or perineural inflammation) may induce more nerve root injury than each factor alone (Figure 7) [44]. The nerve root becomes sensitized by nucleus pulposus material that reaches the epidural space through annular tears [11,37,50,51,52,53,132,133,134,135]. Since the pain threshold has been lowered, the additional mechanical compression on the nerve root may induce an excruciating pain due to the presence of chemical radiculitis resulting from topical exposure of sensory fibers to chemical mediators [44].

### 2.12. Sensitization by Fibrosis

Fibrosis has received considerable attention as a contributor to sciatica and lumbar back pain (LBP) following decompression surgery. Kuslich *et al.* found that spinal nerve roots encased in perineural fibrosis were very sensitive to external stimulation in patients with prior laminectomies undergoing repeat procedures under minimal anesthesia [26]. Neuropathic changes in the spinal sensory potential correlated with postoperative perineural fibrosis in a rat model of laminotomy up to 3 months following surgery [136]. These observations provide direct evidence of nerve root sensitization to pain by perineural fibrosis. While epidural fibrosis commonly results following surgical intervention of the spine, leakage of disc material into the epidural space following an annular tear or surgical intervention can also result in the formation of epidural fibrosis [137,138,139,140,141,142,143,144,145,146,147].

Fransen evaluated the reduction of epidural fibrosis in a group of 396 patients following single level disc herniation and presenting with sciatica often associated with LBP [148,149]. All subjects were operated upon by the same surgeon in the same institution (Clinique du Parc Léopold, Brussels, Belgium) between January 1st 2003 and December 31st 2005. Upon completion of a conventional microdiscectomy, in all patients, the decompressed nerve root and epidural space including the annulus fibrosus were systematically covered with a gel composed of carboxymethylcellulose (CMC) and polyethylene oxide (PEO). Five (5) patients needed reoperations for recurrent herniation, two (2) after less than a week, one after one month, and two (2) within the first year after surgery. In perioperative assessment of the reoperations, there was little or no epidural fibrosis. This facilitated dissection and separation of the nerve root from surrounding tissues. 

### 2.13. Epidural Adhesions

Adhesions themselves are not painful. Epidural fibrosis and subsequent tethering of the nerve root to the disc or pedicle (and thereby compression), may contribute to post-surgical sciatica and lumbar back pain (LBP). However, results of clinical outcome studies attempting to correlate adhesion formation with pain have not been consistent [150,151,152]. Most patients with epidural fibrosis do not develop symptomatic complaints [153]. However, fibrous entrapment of nerve roots may cause sciatica as demonstrated by their release, resulting in immediate relief from sciatica [154]. The pain is thought to result from entrapment of the nerve root by fibrosis resulting in enhanced sensitization in contrast to tethering.

Ido reported seven (7) patients with fibrous adhesive entrapment of lumbosacral nerve roots as a cause of sciatica. Radiographic findings (MRI, myelography and CT myelography) of the patients were negative (no disc herniation or nerve root compression). All seven patients complained of sciatica accompanied by LBP. Differential nerve blocks were effective in relieving sciatica and LBP in these patients. Surgical procedures resulted in the release of the nerve root and creation of space around it. All seven patients experienced complete relief from sciatic pain and LBP immediately after the fibrous sheath was released. During the average follow-up period of 7 years and 2 months, no recurrence of sciatic pain accompanied by LBP was observed [154].

### 2.14. Protection of Epidural Sensory Nerves by Gels

**Figure 8:**
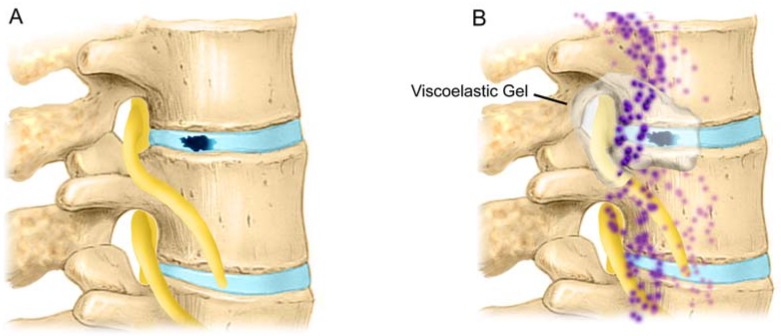
**A**) Disc tear/rupture/annulotomy exposes epidural tissues to the nucleus pulposus. The nucleus pulposus elicits an immune response in the epidural space [121]. **B**) Herniated lumbar disc tissue from symptomatic patients contains elevated levels of TNF- α, nitric oxide (NO), prostaglandin E2 (PGE2); IL-1β, IL-6, IL-8, COX-2, and NOS [103,125,128,179,180] as compared with control disc tissue [41,127,128].

Reduction in fibrin deposition would reduce nerve root entrapment and reduce sensitization to pain [154]. Coating of tissues in the epidural space also reduces postoperative pain following lumbar disc surgery by reducing the interaction of sensory nerves in the lumbar disc and epidural space from cellular and biochemical pain mediators. To be effective, such barrier gels need to be present for a short time to reduce exposure of damaged tissue to macrophages and other inflammatory cells known to stimulate painful nociceptors, and to provide mediators that trigger the development of fibrosis by attracting fibroblasts (Figure 8).

It has been shown in preclinical and clinical studies that coverage of the nerve root with a polyanionic polysaccharide viscoelastic gel such hyaluronic acid (HA), or carboxymethylcellulose (CMC) /polyethylene oxide (PEO) reduces pain and symptoms [155]. Healon® (HA) and Oxiplex® (CMC/PEO) are two such high-molecular-weight polysaccharide gels. The concentration of solids in polysaccharide formulations appears to enhance the mucoadherance and viscoelastic properties that improve tissue coating [7,20,156,157]. A number of preclinical studies demonstrated that HA gel reduces pain and the presence of cellular as well as biochemical pain mediators in rat and canine laminectomy models [6,20,158,159,160,161,162]. Hyaluronic acid gel was also shown to inhibit macrophage migration into the epidural space and release of biochemical pain mediators in the wounds of animals following laminectomy [20,159]. The investigators at the University of California San Diego described a laminectomy model that resulted in a heightened sensitivity to pain [20,158]. Pain reduction by polysaccharide treatment after laminectomy and disc injury in a rat model resulted from reduction in the concentration of the cytokines and inflammatory cell infiltrates that would otherwise occur around the nerve root and the epidural space. 

Exposure of annulus fibrosus and other epidural tissues to inflammatory cells and cytokines results in both nerve sensitization [12] and nerve injury [42]. These cellular and biochemical mediators may be a direct stimulant of LBP by sensitizing peripheral nociceptors [56,52,53]. Hyperalgesic responses can be induced by contact of the nociceptors of sensory nerves with nucleus pulposus that signal LBP directly from the annulus fibrosus and epidural space [38]. Coverage of the annulus and adjacent structures in the epidural space by absorbable viscoelastic gels appears to reduce LBP following surgery by protecting sensory fibers from cellular and biochemical pain mediators. For example, carboxymethylcellulose (CMC) provides tissue coating which protects the surfaces of epidural tissues from topical interaction with cellular and biochemical pain mediators (B); polyethylene oxide (PEO) interacts with the biochemical pain mediators (especially proteins) reducing their topical access to sensory nerves of the annulus fibrosus and epidural space [7]. Gels which protect tissues during healing, thereby reducing their interaction by the interposition of a temporary viscoelastic gel, should provide a useful strategy to reduce both back and leg pain following lumbar disc surgery.

Massie *et al.* concluded that one mode of action for the reduction of pain following surgery with the use of viscoelastic gels is the decreased migration of inflammatory cells into the epidural space by the viscous environment of the gel [6]. Viscoelastic gels would provide a protective tissue coating that decreases fibrosis and shields the nociceptors present on the exposed sensory nerves from pain mediators. The utilization of a mechanical barrier that coats and separates tissues in the lumbar spine provides some measure of surface protection of the sensory nerve against inflammatory mediators that occur as a result of surgery as well as outpouring from the annulotomy site itself. In this mode of action, the tissue coating properties, molecular weight, concentration, and the rheology of the viscoelastic gel are important [7,163,164].

The components of Oxiplex (carboxymethylcellulose, or CMC, and polyethylene oxide, or PEO) separate tissue by providing a viscous coating that prevents migration and attachment of cellular and biochemical pain mediators to covered tissues cells [165,166,167,168,169]. The CMC component allows for gel adherence to tissues [157,170,171]. The PEO component prevents protein deposition [172,173,174,175,176] on the surface of covered tissues. The combination of CMC and PEO allows the gel to remain at the site of application for a period of time, providing a mechanical barrier to protein and cell deposition that could otherwise lead to pain and adhesion formation during the healing process [156,177,178]. 

The data demonstrate that polysaccharide gels that coat healing tissues can protect tissues from cellular and biochemical pain mediators and fibrotic bridges that lead to adhesions during the healing process. Coverage of the annulus and adjacent structures in the epidural space by absorbable viscoelastic gels appears to reduce LBP following surgery by protecting sensory fibers from cellular and biochemical pain mediators. The results of these studies demonstrate that gels which protect tissues during healing, thereby reducing their interaction by the interposition of a temporary viscoelastic gel, should provide a useful strategy to reduce both back and leg pain following lumbar disc surgery.

## Appendix

**Appendix Table 1:** Reports of patients with leg and back pain due to lumbar disc herniation.

Author	No. of Subjects	Conclusion
Spangfort, E.V.	2504	30% continued to have lumbar back pain; 23% continued to have sciatica following discectomy [181].
Pearson, A.M.; *et al.*	775	72% with lumbar back pain after discectomy; sciatica improved more following surgery than lumbar back pain; lumbar back pain improvement only moderately correlated with sciatica improvement [4].
Atlas, S.J.; *et al.*	507	Residual lumbar back pain 1.9; residual sciatica 1.5, 10 years after disc surgery [182].
Dvorak, J.; *et al.*	382	66% continued to have significant lumbar back pain; 45% continued to have sciatica 2-5 years after discectomy [183].
Asch, H.L.; *et al.*	212	Lumbar back pain relief: 77%; sciatica relief: 80% [184].
Loupasis, G.A.; *et al.*	109	Good-excellent relief of lumbar back pain: 80%; good-excellent relief of sciatica: 86% [185]
Silvers, H.R.; *et al.*	104	Relief of lumbar back pain: 76%; relief of sciatica: 89% following discectomy [186].
Lewis, P.J.; *et al.*	100	13% had greater lumbar back pain than sciatica at baseline [187].
Weber, H.	45	17% of 45 subjects no lumbar back pain; 41% of 36 subjects no sciatica, 4 years after discectomy [188].
Toyone, T.; *et al.*	40	Lumbar disc herniation might be a possible cause of lumbar back pain; potential sensory pathways described for discogenic pain due to disc herniation [8].
Korhonen, T.; *et al.*	21	Lumbar back pain relief 51%; sciatica relief 68% following novel drug therapy [189].
Silvers, H.R.; *et al.*	15	Relief of lumbar back pain: 77%; relief of sciatica: 85% following discectomy [190].
Osterman; *et al.*	58	Leg pain baseline VAS score 9/100, back pain VAS score 7/100. Only leg pain superior at 6 weeks [191].
Peul; *et al.*	283	Baseline VAS score; leg pain 17.7/100, back pain 11.3/100. 81% disappearance of symptoms at 8 weeks [192,193].

**Appendix Table 2:** Biochemical pain mediators.

Mediator	Description / Complete Name	Activity	Source / Location	Reference
Cytokines/Growth Factors (Signaling Substances)				
COX	Cyclooxygenase	The induction of cyclooxygenase-2 (PTGS2/COX2) by IL-1β in the central nervous system contributes to inflammatory pain hypersensitivity. Mediates prostaglandin E2 (PGE2).	Central nervous system.	Walsh [194]
FGF2 or FGFb	Fibroblast growth factor, basic	Involved in angiogenesis, wound healing, and embryonic development. Heparin-binding proteins and interactions with cell-surface associated heparan sulfate proteoglycans have been shown to be essential for FGF signal transduction. FGF2s are key players in the processes of proliferation and differentiation of wide variety of cells and tissues.	Secreted by leukocytes.	Weiler [126]
GAP-43	Growth-associated protein 43	A marker for axonal growth. GAP-43 expression is up regulated with TNF-α at high concentrations in both NGF and glial cell-line derived neurotrophic factor-sensitive (GDNF) neurons in response to neuronal injury, and importantly, to a greater extent in nerve growth factor (NGF)-sensitive neurons than in GDNF-sensitive neurons. This suggests that NGF-sensitive neurons have the potential to extend their axons in a response to neuronal injury more markedly than GDNF-sensitive neurons. Almost all of the disc-innervating neurons are NGF-sensitive type and axonal injury or stimulus to the dorsal root ganglion may cause disc innervation.	Central nervous system.	Aoki [195]
GM-CSF	Granulocyte macrophage colony stimulation factor	GM-CSF stimulates stem cells to produce granulocytes (neutrophils, eosinophils, and basophils) and monocytes. Monocytes exit the circulation and migrate into tissue, whereupon they mature into macrophages. It is thus part of the immune/inflammatory cascade.	Secreted by macrophages, T cells, mast cells, endothelial cells and fibroblasts. Intervertebral discs.	Weiler [126]
IGF-1	Insulin-like growth factor	IGF-1 regulates cell growth and development, especially in nerve cells.	IGF-1 is produced primarily by the liver as an endocrine hormone as well as in target tissues.	Weiler [126]
IL-1	Interleukin-1	Mediator of the peripheral inflammatory response including co-stimulation of APCs and T cells, inflammation and fever, acute phase response, hematopoiesis. Synthesized and released at various disease states.	Secreted by macrophages, B cells, monocytes, dendritic cells.	Ohtori [58]
		IL-1 treatment has been linked to increases in proinflammatory factor production via activation of NF-κβ, a major regulator of inflammatory cytokine-inducible genes. IL-1 pretreatment has been shown to stimulate the production of the reactive oxygen species nitric oxide (NO) and increased synthesis of nitric oxide synthase (NOS). IL-1 has been shown to decrease proteoglycan synthesis and increase matrix catabolism via up regulated matrix metalloproteases (MMP) activity in osteoarthritic cartilage.		Walsh [194]
IL-1β	Interleukin-1β	Stimulates the production of nerve growth factor (NGF) and mRNA expression. Stimulates degradation of the extracellular matrix and induce changes to the biochemical properties of the discs, notably the loss of proteoglycans. Up regulates the expression of NGF and causes inflammatory pain. Proinflammatory cytokines are expressed by animal intervertebral discs (IVDs), human IVDs or herniated IVDs.	Secreted primarily by macrophages; all nucleated cells.	Abe [40]
		Inflammatory signaling substance.		Brisby [47]
		Potent pain generating mediator. Stimulates PEG_2_, COX2, and matrix metalloproteases (MMPs) production.		Omoigui [196]
IL-6	Interleukin-6	Mediator of the peripheral inflammatory response; acute phase response, B cell proliferation, thrombopoiesis, synergistic with IL-1 and TNF on T cells. Synthesized and released at various disease states.	Secreted by macrophages, activated TH2-cells, B cells, astrocytes, endothelial cells, hepatocytes and adipocytes.	Ohtori [58]
		Inflammatory signaling substance.		Brisby [47]
		Potent pain generating mediator. Plays an active role in inflammation and immunology. IL-6 is the primary chemical mediator involved in bone inflammation and bone pain. IL-6 production is increased by IL-1β and TNF- α.		Omoigui [196]
IL-8	Interleukin-8	Inflammatory signaling substance. Chemoattractant for neutrophils and T cells.	Secreted by macrophages, lymphocytes, epithelial cells, and endothelial cells.	Brisby [47]
IL-10	Interleukin-10	One of the natural anti-inflammatory cytokines. Inhibits cytokine production, promotes B cell proliferation and antibody production, suppresses cellular immunity, mast cell growth.	Secreted by macrophages, monocytes, TH2-cells, CD8+ T cells, mast cells, B cell subset. Intervertebral disc.	Weiler [126]
				Omoigui [196]
NGF	Nerve growth factor	Promotes neurite outgrowth and neural cell survival. Up regulated by proinflammatory cytokines. May play a role in the collateral sprouting of sensory axons, neural survival and regulation of nociceptive sensory neurons. Promotes nerve in-growth in to the intervertebral disc (IVD). NGF has been reported to be synthesized and released from innervated target tissues and to induce nerve in-growth in the target tissue. Directly modulates the function of nociceptive sensory neurons, resulting in pathological pain. May contribute to tissue repair. Inflammatory mediator by proliferation and/or activation of lymphocytes, eosinophiles, or mast cells. NGF produced by the intervertebral disc (IVD) cells, or by repair tissue, may sensitize these NGF-sensitive neurons, possibly resulting in discogenic pain. NGF expression level to cytokines may be dependent on the grade of disc degeneration. Expression of TrkA in IVD cells may suggest that NGF can affect IVD cells in a paracrine fashion, such as by the induction of transforming growth factor β, or by preventing apoptosis. The majority of disc innervating dorsal root ganglion neurons were NGF-sensitive neurons (rat model). Nociceptive dorsal root ganglion neurons were shown to include 2 types: nerve growth factor (NGF)-sensitive and glial cell line-derived neurotrophic factor (GDNF)-sensitive neurons.	Produced in the peripheral and central nervous systems (CNS). Can be produced by cells outside the CNS (e.g., lymphocytes, mast cells, keratinocytes, fibroblasts) IVD tissue (receptor location) show expression of NGF. Rounded chronrocyte-like cells in the annulus fibrosis were immunohistochemically positive for NGF. Nucleus pulposus cells may produce more NGF that annulus fibrosis cells.	Abe [40]
NO	Nitric oxide	Inflammatory signaling substance induced by IL-1β. Autocrine regulator of fibroblast collagen synthesis and contraction. Has been shown to decrease proteoglycan synthesis and increase matrix catabolism via up regulated matrix metalloproteases (MMP) activity in osteoarthritic cartilage. NO can activate the mitogen-regulated kinase (mitogen-activated protein kinase) pathways, ERK1/2 and JNK leading to increased activity of phospholipase A2 and cyclooxygenase-s (COX2) and ultimately prostaglandin E2 (PEG2) production. Enhanced or maintained production of NO by annular cells may be desirable because NO is an autocrine regulator of fibroblast collagen synthesis and contraction and may therefore enhance wound healing. Glucosamine can reduce production of NO by nucleus cells but not annulus cells.	Secreted by monocytes, macrophages, and neutrophils. Produced by nucleus pulposus cells and annulus fibrosis cells.	Walsh [194]
PGE2	Prostaglandin E2 [23, 24]	PGE2 is a direct vasodilator and it inhibits the release of noradrenaline from sympathetic nerve terminals. Sensitize spinal neurons to pain. Pyrogenic.	Secreted in microvessels by the endothelium.	Weiler [126]
		PGE2 has been shown to decrease proteoglycan synthesis and increase matrix catabolism via up regulated MMP activity in osteoarthric cartilage. Induced by IL-1β. Glucosamine can reduce production of PEG2 by nucleus cells but not annulus cells. Mediated by phospholipase and cyclooxygenase-2 (COX2).		Walsh [194]
PLA2	Phospholipase A2	Upstream regulator of the inflammatory process. Regulated by phosphorylation and calcium concentrations. Mediates PGE2.		Weiler [126]
TGF-β	Transforming growth factor- β	Anti-inflammatory, promotes wound healing, inhibits macrophage and lymphocyte proliferation. Provides a strong inhibitory signal to nitric oxide synthase (NOS).	Activated Th1 cells (T-helper) and natural killer (NK cells)	Weiler [126]
TNF-α	Tumor necrosis factor-α	Stimulates the production of nerve growth factor (NGF) and mRNA expression. Stimulate degradation of the extracellular matrix and induce changes to the biochemical properties of the discs, notably the loss of proteoglycans. Up regulates the expression of NGF and causes inflammatory pain.Up regulation of NGF mRNA expression by TNF-α is higher in nucleus pulposus cells than in annulus fibrosis cells. Proinflammatory cytokines are expressed by animal intervertebral disc (IVD), human IVDs or herniated IVDs.	Secreted by macrophages, lymphoid cells, mast cells, endothelial cells, cardiac myocytes, adipose tissue, fibroblasts and neuronal tissue.	Abe [40]
		TNF-α is expressed in the human degenerated intervertebral disc, especially in symptomatic discs. TNF-α is thought to be involved in the mechanism of nucleus pulposus-induced nerve root injury. TNF inhibitors (etanercept and infliximab) have been shown to be effective in preventing pathologic changes in nucleus pulposus-induced nerve injury. Or mechanical and thermal hyperplasia. On the other hand, and *in vitro* study showed that TNF-α inhibited axonal growth of dorsal root ganglion (DRG) neurons cultured as an organ culture. It is speculated that TNF-α secreted from pathologic discs can injure adjacent dorsal root ganglions (DRG) and also induce nerve in-growth from the injured dorsal root ganglions into the discs.		Aoki [195]
		After peripheral nerve injury TNF expression is up regulated in endoneurial macrophages and Schwann cells, resulting in pain. TNF is axonally transported from the inflammatory site to the dorsal root ganglion (DRG) neurons and the spinal chord dorsal horn where it correlates with the expression of TNF receptors Type 1 and Type 2, which does not usually exist in dorsal root ganglion (DRG) neurons.	Schwann cells.	Sakuma 2007 [124], Ohtori [58]
		Synthesized as a precursor protein that is proteolytically processed at the cellular surface via the TNF-α converting enzyme (TACE). The main effects of TNF-α are 1) up regulation of matrix metalloproteinase activity and gene expression, 2) stimulation of other potent cytokines like IL-1, IL-6, IL-8 and prostaglandin E2 (PGE2) 3) stimulation of cell migration and altered endothelial permeability, 4) decrease of collagen and proteoglycan synthesis, 5) development of inflammatory hyperalgesia. An important cytokine in nerve root injury because it is known to be neurotoxic and to induce axonal and myelin injury, intravascular coagulation and increased vascular permeability.	Synthesized in the nuclear and anular disc regions.	Weiler [126]
**Neuropeptides (Ligands)**				
VEGF	Vascular endothelial growth factor	A heparin binding, homodimeric glycoprotein. Several cytokines and growth factors including epithelial growth factor (EGF) and TGF-β up regulate VEGF mRNA. IL-1α and prostaglandin E2 (PGE2) also induce VEGF expression in cultured synovial fibroblasts, suggesting a role for inflammatory mediators in VEGF induction of inflammatory angiogenesis. VEGF plays an important role in the initiation of angiogenesis through stimulation of the formation of new blood vessels, lumen formation and endothelial migration. VEGF also induces Pas resulting in the generation of plasmin. In turn, plasmin activates matrix metalloproteases (MMP), which are required for matrix degradation that occurs during the resorption process of HD. VEGF expression is strongly up regulated by both macrophages and intervertebral disc tissues using co-culture conditions. Macrophages or disc cells cultured alone produce low levels of VEGF protein. VEGF appears to be mediated through a TNF-α pathway as this effect was abrogated by the use of a TNF-α neutralizing antibody.	Endothelial cell-specific mitogen. Expressed in human herniated disc tissues.	Haro [119]
CGRP	Calcitonin-gene related peptide	Marker for nerve growth factor (NGF)-sensitive neurons. Strong correlation between the expression of CGRP and nerve growth factor receptors, and between the expression of isolectin B4-binding glycoprotein and the glial cell-line derived neurotrophic factor receptor.	Produced in nervous tissue. Receptors to CGRP are expressed throughout the body.	Aoki [195]
		Sensory nerve transmitter related to pain.	CGRP immunoreactive nerve fibers are present within lumbar Intervertebral discs.	Ohtori [58]
(PK) C	Protein kinase C	Highly regulated protein modifier. Regulate cellular pathways, especially signal transduction.		Yaksh [197]
Proteoglycans		Suppresses nerve in-growth into the intervertebral disc (IVD). Change in proteoglycan content of degenerated discs has been suggested to be a contributing factor in IVD innervation.	Intervertebral discs.	Abe [40]
**Neurotransmitters (Receptors)**				
AMPA	Alpha-amino-3-hydroxy-5-methyl-4-isoxazolepropionic acid	A compound that is a specific agonist for the AMPA receptor, where it mimics the effects of the neurotransmitter glutamate. An ionotropic receptor for glutamate. Contributes to postsynaptic action. Repetitive small afferent input (as that which occurs after tissue injury) will evoke spinal glutamate release. The spinal delivery of agonists of the ionotrophic glutamate receptors (NMDA/AMPA) evokes potent spontaneous and subsequent thermal hyperalgesia and tactile allodynia.	Central nervous system (CNS) / Dorsal grey matter.	Yaksh [197]
Glutamate	Glutamate	Glutamate is stored in vesicles at chemical synapses. Nerve impulses trigger release of glutamate from the pre-synaptic cell. In the opposing post-synaptic cell, glutamate receptors, such as the n-methyl-D-aspartate (NMDA) receptor, bind glutamate and are activated. Most abundant excitatory neurotransmitter in the mammalian nervous system.	Carboxylate anions or salts of glutamic acid.	Yaksh [197]
NK-1	Neurokinin-1 receptor	Receptor for Substance P.	Distributed in the dorsal root ganglion and the spinal dorsal horn.	Ohtori [58]
NMDA	n-methyl-D-aspartate	An ionotropic receptor (ligand-gated ion channel) for glutamate. Contribute to postsynaptic action. Repetitive small afferent input (as that which occurs after tissue injury) will evoke spinal glutamate release. The spinal delivery of agonists of the ionotrophic glutamate receptors (NMDA/AMPA) evokes potent spontaneous and subsequent thermal hyperalgesia and tactile allodynia.	Central nervous system (CNS) / Dorsal grey matter.	Yaksh [197]
P (sP)	Substance P	Sensory nerve transmitter related to pain.	SP immunoreactive nerve fibers are present within lumbar intervertebral discs.	Ohtori [70]
p75^NGFR^	p75^NGFR^	Low affinity nerve growth factor (NGF) receptor.	Localized in annulus fibrosis and nucleus pulposus cells.	Abe [40]
TrkA	Transmembrane tyrosine kinase (“TrackA”)	High affinity nerve growth factor (NGF) receptor. Expression of TrkA in intervertebral disc (IVD) cells may suggest that NGF can affect intervertebral disc cells in a paracrine fashion, such as by the induction of transforming growth factor β (TGF-β), or by preventing apoptosis.	Localized in annulus fibrosis and nucleus pulposus cells (within the cell cytoplasm).	Abe [40]

Inflammation and the inflammatory response is the underlying source of leg pain and back pain. The biochemical mediators of inflammation include cytokines, growth factors, neuropeptides and neurotransmitters [196]. The following table includes biochemical inflammatory pain mediators identified in the epidural space in patients with lumbar back pain [40,58,70,124,195,196].
